# HIST1H1B Promotes Basal-Like Breast Cancer Progression by Modulating CSF2 Expression

**DOI:** 10.3389/fonc.2021.780094

**Published:** 2021-10-22

**Authors:** Ruocen Liao, Xingyu Chen, Qianhua Cao, Yifan Wang, Zhaorui Miao, Xingyu Lei, Qianjin Jiang, Jie Chen, Xuebiao Wu, Xiaoli Li, Jun Li, Chenfang Dong

**Affiliations:** ^1^ Department of Pathology and Pathophysiology, and Department of Colorectal Surgery and Oncology, Key Laboratory of Cancer Prevention and Intervention, Ministry of Education, The Second Affiliated Hospital, Zhejiang University School of Medicine, Hangzhou, China; ^2^ Zhejiang Key Laboratory for Disease Proteomics, Zhejiang University School of Medicine, Hangzhou, China; ^3^ Cancer Institute of Integrative Medicine, Zhejiang Academy of Traditional Chinese Medicine, Tongde Hospital of Zhejiang Province, Hangzhou, China; ^4^ Department of Pathophysiology, Gannan Medical University, Gannan, China; ^5^ R&D Department of Hangzhou, Abcam Plc, Hangzhou, China

**Keywords:** HIST1H1B, CSF2, DNA methylation, copy number variant (CNV), basal-like breast cancer (BLBC)

## Abstract

**Background:**

Basal-like breast cancer (BLBC) is associated with a poor clinical outcome; however, the mechanism of BLBC aggressiveness is still unclear. It has been shown that a linker histone functions as either a positive or negative regulator of gene expression in tumors. Here, we aimed to investigate the possible involvement and mechanism of HIST1H1B in BLBC progression.

**Experimental design:**

We analyzed multiple gene expression datasets to determine the relevance of HIST1H1B expression with BLBC. We employed quantitative real-time PCR, transwell assay, colony formation assay, and mammosphere assay to dissect the molecular events associated with the expression of HIST1H1B in human breast cancer. We studied the association of HIST1H1B with CSF2 by ChIP assay. Using tumorigenesis assays, we determine the effect of HIST1H1B expression on tumorigenicity of BLBC cells.

**Results:**

Here, we show that the linker histone HIST1H1B is dramatically elevated in BLBC due to HIST1H1B copy number amplification and promoter hypomethylation. HIST1H1B upregulates colony-stimulating factor 2 (CSF2) expression by binding the CSF2 promoter. HIST1H1B expression promotes, whereas knockdown of HIST1H1B expression suppresses tumorigenicity. In breast cancer patients, HIST1H1B expression is positively correlated with large tumor size, high grade, metastasis and poor survival.

**Conclusion:**

HIST1H1B contributes to basal-like breast cancer progression by modulating CSF2 expression, indicating a potential prognostic marker and therapeutic target for this disease.

## Introduction

The linker histone H1, interacts with the DNA entering and exiting the nucleosomal core particle (1–3), which displays much higher sequence variability between different species than the evolutionary extremely conserved core histones (4). It has been showed that like core histones, a linker histone functions as either a positive or negative regulator of gene expression *in vivo* (5). HIST1H1B (H1.5) is one of eleven variants in linker histone family in human (6), and recently more studies have focused on its disease processes (7, 8). HIST1H1B gene has been found to be frequently mutated in colorectal cancer and follicular lymphomas (9). Additionally, Mazdak *et al.*’s study has showed that the malignant leiomyosarcomas displays a 5.6 times higher HIST1H1B expression than the benign leiomyoma samples, and HIST1H1B overexpression contributes to tumor aggressiveness (8). However, many mechanisms and functions of HIST1H1B are still unknown.

Colony-stimulating factor 2 (CSF2), also known as granulocyte macrophage-colony stimulating factor (GM-CSF), is a cytokine functioning as a cytokine that stimulates the growth and differentiation of stem cells (10). CSF2 have been proved to be closely linked with poor prognosis in some tumors (11, 12). In this study, we show that HIST1H1B expression occurs specifically in BLBC and predicts poor prognosis. We also provide evidence that HIST1H1B enhances tumorigenesis through modulating CSF2 expression in BLBC.

## Methods

### Plasmids and Antibodies

Human HIST1H1B genes were amplified from MDA-468 cDNA libraries, and sub-cloned in plvx-puro. HIST1H1B shRNA was purchased from MISSION shRNA at Sigma-Aldrich (St Louis, MO). Antibodies against HIST1H1B were purchased from Affinity Biosciences.

### Cell Culture

SUM159, Hs578T and BT20cells were maintained in DMEM/F12 supplemented with 10% fetal bovine serum (FBS). BT549 cells were grown in RPMI1640 containing 10% FBS. MDA-MB468 cells were cultured in Leibovitz’s L-15 medium with 10% FBS. All cell lines were maintained in a humidified 5% CO2 atmosphere at 37°C. For establishing stable transfectants with HIST1H1B expression or knockdown of HIST1H1B expression, BLBC cells were transfected with plvx-HIST1H1B and HIST1H1B shRNA, and the transfected cells were selected in puromycin (300ng/mL) for 3 weeks.

### Quantitative Real-Time PCR

Total RNA was extracted from cells using Trizol (Accurate Biology) according to the manufacturer’s instructions. Reverse transcription was carried out with the Evo M-MLV RT Premix for qPCR (Accurate Biology). Specific quantitative real-time PCR was carried out using the SYBR Green Premix Pro Taq HS qPCR Kit according to the manufacturer’s protocol (AG). Gene expression level was normalized to actin level as an internal control, and the results were representative of at least three independent experiments.

### Western Blot Analysis

The protein was extracted from each sample using RIPA buffer containing protease inhibitors. Protein concentration was examined by using Bradford assay Kit (Ddbio science) with BSA as standard. Adjusted all protein samples to the same concentration, and mixed with loading buffer, and then electrophoresed on 10% sodium dodecyl sulfate-polyacrylamide (SDS-PAGE) gels. Separated proteins were transferred to polyvinylidene fluoride (PVDF) membranes. The membranes were blocked with 5% fat-free milk for 1 h, and then were incubated with diluted primary antibody in 5% w/v BSA, 1X PBS, 0.1% Tween^®^ 20 at 4°C with gentle shaking, overnight, and with secondary antibodies for 1 h. The blots were visualized using ECL assay.

### Chromatin Immunoprecipitation

ChIP assays were performed as described previously (13, 14). The following primers were used for ChIP assays: 5’-TGTCGGTTCTTGGAAAGGTTCA -3′and 5’- TGTGGAATCTCCTGGCCCTTA for the CSF2 promoter (15). The cells were prepared for ChIP assay as described recently (14).

### Colony Formation Assay

Colony formation assay was carried out using double-layer soft agar in 24-well plates with a top layer of 0.35% agar and a bottom layer of 0.7% agar. Cells were seeded in 24-well plates and cultured at 37°C for 14-21 days, and the colonies were counted as described previously (16).

### Migration and Invasion Assays

Migration and invasion assays were carried out as described previously (17). All experiments were repeated at least twice in triplicate. Statistical analysis was performed using the Student’s t-test; a p-value of <0.05 was considered significant.

### Tumorigenesis Assay

Animal experiments were performed according to the approved procedures by the Institutional Animal Care and Use Committee at Zhejiang University. To test the effect of HIST1H1B on *in vivo* tumorigenesis, female SCID mice (5-6 wks old) were injected with 5×10^6^ exogenous HIST1H1B knockdown cells in the left flank and vector control cells in the right flank. The tumor size was examined every 2 to 4 days for 30 days, and tumor weight were measured.

### Statistical Analysis

Results are expressed as mean ± SD or SEM as indicated. Comparisons were made by the two-tailed Student’s t-test or one-way ANOVA. Correlations were analyzed by Pearson’s correlation method and Spearman’s rank correlation test. Survival curves were plotted using the Kaplan-Meier method, and differences were analyzed by the log-rank test. In all statistical tests, p < 0.05 was considered statistically significant.

## Results

### HIST1H1B Expression Is Upregulated in Breast Cancer

**Figure 1:**
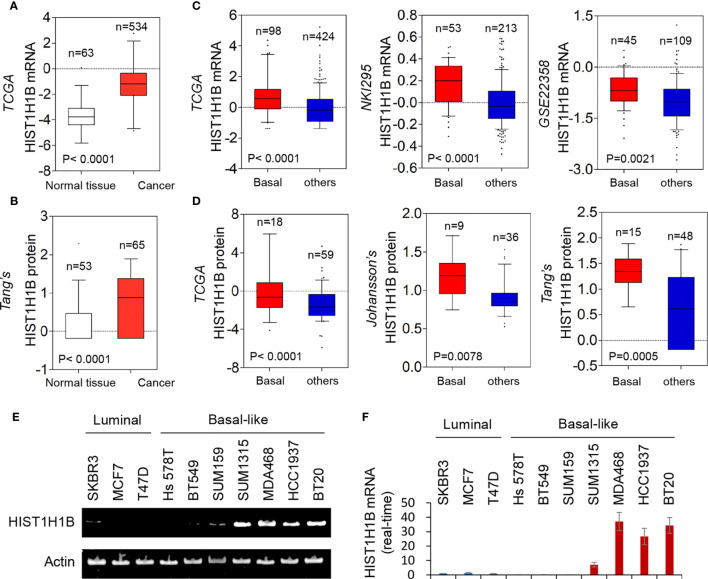
HIST1H1B expression is upregulated in breast cancer. **(A)** Box-plots indicated HIST1H1B mRNA expression in breast cancer from TCGA. **(B)** Box-plots indicate HIST1H1B protein expression in different subtypes of breast cancer from the Tang's dataset. **(C)** Box-plots indicated HIST1H1B mRNA expression in different subtypes of breast cancer from three datasets (NKI295, TCGA and GSE22358). **(D)** Box-plots indicated HIST1H1B protein expression in different subtypes of breast cancer from three datasets (TCGA, Johansson's and Tang's). **(E, F)** Expression of HIST1H1B mRNA was analyzed by either quantitative real-time PCR **(E)** or semi­ quantitative RT-PCR **(F)** in a representative panel of breast cancer cell lines.

We recently reported that several molecules, aldo-keto reductase 1 member B1 (AKR1B1), fructose-1,6-biphosphatase (FBP1), and urine diphosphate–galactose ceramide galactosyltransferase(UGT8) were associated with breast cancer aggressiveness. To find other determinants for breast cancer aggressiveness, we analyzed several publicly available gene expression datasets (TCGA, NKI295 and GSE22358), which contain 597, 266 and 154 breast cancer patients, respectively. Notably, HIST1H1B mRNA expression was significantly higher in breast cancer than in normal breast tissues (**Figure 1A**). Additionally, HIST1H1B mRNA expression is remarkably upregulated in basal-like breast cancer (BLBC) compared with other subtypes (**Figure 1C**). Consistent with these observations, HIST1H1B protein expression also was significantly higher in breast cancer than in normal breast tissues, and in BLBC than other subtypes by proteogenomic analysis of multiple datasets (TCGA, Johansson’s and Tang’s) that contains 77, 45 and 63 breast tumor samples, respectively (**Figures 1B, D**). To evaluate the HIST1H1B expression in different breast cell lines, we analyzed the HIST1H1B expression by either semi-quantitative RT-PCR or quantitative real-time PCR in 3 luminal and 7 BLBC cell lines. We found that HIST1H1B expression was much higher in BLBC cells than in luminal cells (**Figures 1E, F**). These findings indicate that HIST1H1B overexpression positively correlates with the breast cancer, especially BLBC subtype.

### HIST1H1B Expression Is Upregulated Due to Amplified HIST1H1B Copy Number and Low Promoter Hypomethylation of HIST1H1B

**Figure 2:**
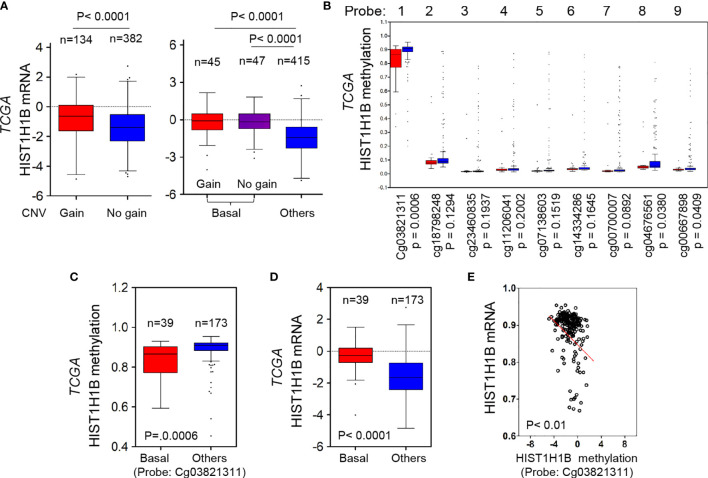
HIST1H1B overexpression associates with HIST1H1B's copy number amplification and promoter hypomethylation. **(A)** Box-plots indicated the association of HIST1H1B expression with its copy number status (gain or no gain) in breast cancer from the TCGA dataset. **(B)** Analysis of HIST1H1B methylation using multiple 450 K probes in different subtypes of breast cancer from the TCGA dataset. **(C)** Box-plots indicated HIST1H1B methylation in different subtypes of breast cancer from the TCGA dataset. **(D)** Box-plots indicated HIST1H1B mRNA in different subtypes of breast cancer from the TCGA dataset. **(E)** Analysis of the TCGA dataset for HIST1H1B mRNA expression and HIST1H1B methylation. The relative level of HIST1H1B mRNA was plotted against that of and HIST1H1B methylation.

Copy Number Variants (CNVs) of protein coding genes are closely related with gene expression changes. To investigate the effect of CNVs on HIST1H1B expression, we analyzed copy number alterations of breast cancer in the TCGA dataset from breast cancer tissues. We observed that cases with HIST1H1B copy number amplification had significantly higher HIST1H1B expression than the one with no amplification, supporting that HIST1H1B copy number amplification positively correlates with HIST1H1B overexpression in breast cancer. Consistently, in most cases copy number amplification in HIST1H1B and its resultant HIST1H1B overexpression were positively correlated with BLBC (**Figure 2A**). These data strongly support the association of HIST1H1B copy number amplification with HIST1H1B overexpression and BLBC subtype.

Given that many tumors with HIST1H1B overexpression didn’t have copy number amplification in HIST1H1B, we reasoned that other mediators might involve this event. Aberrant DNA methylation controls gene expression, and thus it usually functions as a transcriptionally regulating component (18). To evaluate whether DNA methylation affects HIST1H1B levels within breast tumors, we then analyzed methylation and expression datasets from breast cancer generated by TCGA. The correlation between HIST1H1B mRNA levels assessed by gene expression microarray and HIST1H1B methylation measured by 450 K Infinium microarray was analyzed. As expected, HIST1H1B promoter methylation in BLBC had a decreased trend in most patients, and the regions (450 K probes 1, 8 and 9) in BLBC showed significantly less methylations than in the other subtypes (**Figure 2B**). Remarkably, HIST1H1B promoter methylation inversely correlated with HIST1H1B mRNA levels (**Figures 2C–E**). These data indicate that hypomethylation of HIST1H1B promoter is critical for HIST1H1B overexpression.

### HIST1H1B Expression Enhances Breast Cancer Cell Migration and Invasion

**Figure 3:**
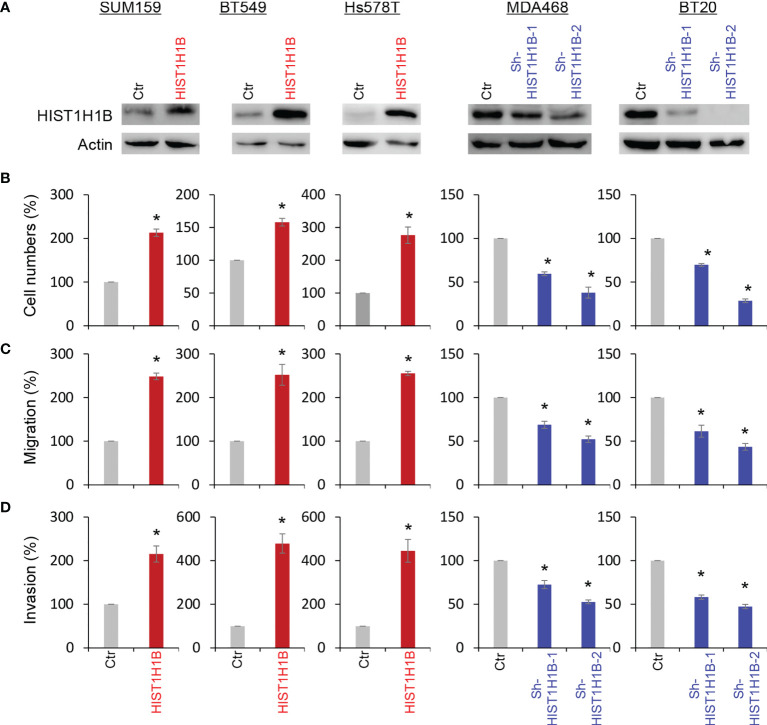
HIST1H1B expression promotes breast cancer cell proliferation, migration and invasion. **(A)** Expression of HIST1H1B was examined by Western blotting in SUM159, BT549 and Hs578T cells with stable empty vector or HIST1H1B expression as well as MDA468 and BT20 cells with stable empty vector or knockdown of HIST1H1B expression. **(B)** Proliferation of SUM159, BT549 and Hs578T cells with stable empty vector or HIST1H1B expression (left panel) as well as MDA468 and BT20 cells with stable empty vector or knockdown of HIST1H1B expression (right panel) was analyzed by cell-count assays for 4 days. Data are shown as a percentage of control cells (mean ± SD in two independent experiments) *p < 0.01 by Student's t-test. **(C, D)** Migration **(C)** and invasiveness **(D)** of SUM159, BT549 and Hs578T cells with stable empty vector or HIST1H1B expression (left panel) as well as MDA468 and BT20 cells with stable empty vector or knockdown of HIST1H1B expression (right panel) were analyzed. The percentage of migratory and invasive cells is presented in the bar graph (mean ± SD in three separate experiments). *p < 0.01 by Student's t-test.

To access the molecular functions and mechanisms of HIST1H1B, we established stable transfectants with empty vector or HIST1H1B expression in SUM159, BT549 and Hs578T cells and clones with empty vector or knockdown of HIST1H1B expression in MDA-468 and BT20 Cells (**Figure 3A**). First, we analyzed the effect of HIST1H1B expression on breast cancer cell proliferation, and found that HIST1H1B expression led to a significant increase in the proliferation of SUM159, BT549 and Hs578T cells, whereas knockdown of HIST1H1B expression caused a remarkable decrease in MDA-468 and BT20 cell proliferation (**Figure 3B**). Analysis of the effect of HIST1H1B expression on breast cancer cell migration and invasion showed that HIST1H1B expression led to significant increase in the migration and invasion of SUM159, BT549 and Hs578T cells, whereas knockdown of HIST1H1B expression markedly repressed the migration and invasion of MDA-468 and BT20 Cells (**Figures 3C, D**). These data suggest that HIST1H1B expression plays a key role in the proliferation, migration, and invasion of breast cancer cells.

### HIST1H1B Expression Upregulates CSF2 Expression

**Figure 4:**
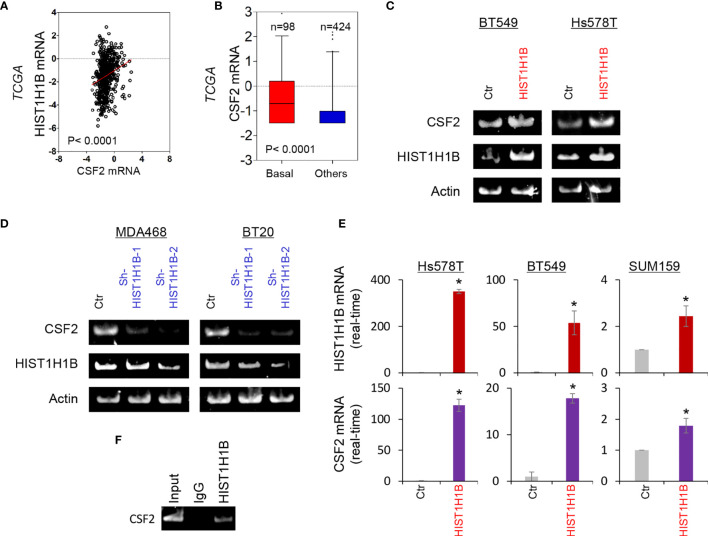
HIST1H1B promotes CSF2 expression. **(A)** Analysis of the TCGA dataset for the expression of HIST1H1B and CSF2. The relative level of HIST1H1B is plotted against that of CSF2. **(B)** Box-plots indicated CSF2 mRNA expression in different subtypes of breast cancer from the TCGA dataset. **(C, D)** Expression of HIST1H1B and CSF2 was analyzed by semi-quantitative RT-PCR in SUM159, Hs578T and BT549 cells with empty vector or HIST1H1B expression **(C)** as well as MDA-MB468 and BT20 cells with stable empty vector or knockdown of HIST1H1B expression **(D)**. Data are shown as mean ± SD based on three independent experiments. *P < 0.05 by Student's t test. **(E)** Expression of HIST1H1B and CSF2 mRNA was examined by quantitative real-time PCR in Hs578T, BT549 and SUM159 cells with stable empty vector or HIST1H1B expression. **(F)** ChiP analysis for binding of HIST1H1B to the CSF2 promoter in SUM159 cell.

To investigate potential molecular mechanisms of HIST1H1B in breast cancer, we performed coexpression analysis of HIST1H1B with other genes in the TCGA dataset. Several genes, such as IDO1, IL10 and CSF2, have been noticed to have positive correlation with HIST1H1B, and we chose CSF2 that plays critical roles in breast cancer aggressiveness to further analyze its links with HIST1H1B. We showed that HIST1H1B expression positively correlated with CSF2 expression (**Figure 4A**). Additionally, analysis of CSF2 expression in different subtypes of breast cancer showed that similar to HIST1H1B, CSF2 expression was significantly upregulated in BLBC in the TCGA dataset (**Figure 4B**). We then explored the causal relationship between HIST1H1B and CSF2 by semi-quantitative RT-PCR or quantitative real-time PCR. Strikingly, knockdown of HIST1H1B expression caused a decrease, whereas HIST1H1B expression resulted in an increase, in CSF2 expression (**Figures 4C–E**). It has been reported that some linker histones are associated with transcriptional activity of genes (5). To determine whether HIST1H1B regulated CSF2 expression by targeting CSF2 promoter, we performed chromatin immunoprecipitation (ChIP) assay in in SUM159 cells. Strikingly, HIST1H1B directly bound to the CSF2 promoter (**Figure 4F**). These data indicate a critical role of HIST1H1B in regulating CSF2 expression.

### HIST1H1B Promotes Tumorigenicity of Breast Cancer

**Figure 5:**
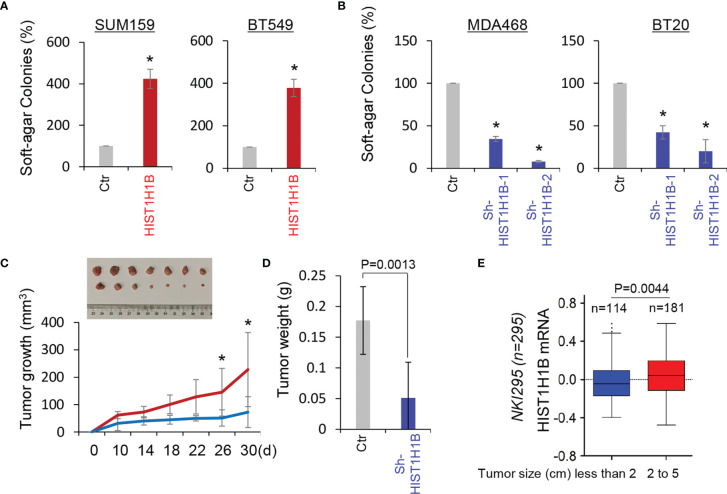
Knockdown of HIST1H1B expression suppresses tumorigenicity in vitro and in vivo. **(A, B)** Soft-agar assay was performed using SUM159 and BT549 cells with stable empty vector or HIST1H1B expression **(A)** as well as MDA468 and BT20 cells with stable empty vector or knockdown of HIST1H1B expression **(B)**. Data are presented as the percentage of vector cell lines (mean ± SD in three separate experiments). *p < 0.01 by Student's t-test. **(C, D)** MDA468 cells with stable empty vector or knockdown of HIST1H1B expression were injected into the mammary fat pad of SCID mice. Tumor growth **(C)** was recorded every two days, and tumor weights **(D)** were measured. Data are presented as mean ± SEM from seven mice. *p < 0.05. **(E)** Box-plots indicated HIST1H1B expression in different tumor sizes of breast cancer from the NKI295 dataset. Comparisons are made using the two-tailed Student's t-test.

Given the tight association of HIST1H1B with BLBC, we assessed the functional role of HIST1H1B in the *in vitro* tumorigenicity using the soft-agar assay. HIST1H1B expression led to a remarkable increase of colonies in SUM159 and BT549 cells, whereas knockdown of HIST1H1B expression resulted in an apparent decrease of colony-formation in MDA-468 and BT20 cells (**Figures 5A, B**). We then evaluated the *in vivo* tumorigenicity using tumor xenograft models. Markedly, MDA-468 cells with stable knockdown of HIST1H1B expression caused reduced tumor growth *in vivo* (**Figures 5C, D**). To support the clinical association of HIST1H1B expression with tumor growth, we evaluated the link of HIST1H1B expression with tumor size in the NKI295 dataset. Patients were separated into two groups based on the primary tumor size. Significantly, high HIST1H1B expression was correlated with a larger tumor size (**Figure 5E**). These data indicate the importance of HIST1H1B expression for tumorigenicity.

**Figure 6:**
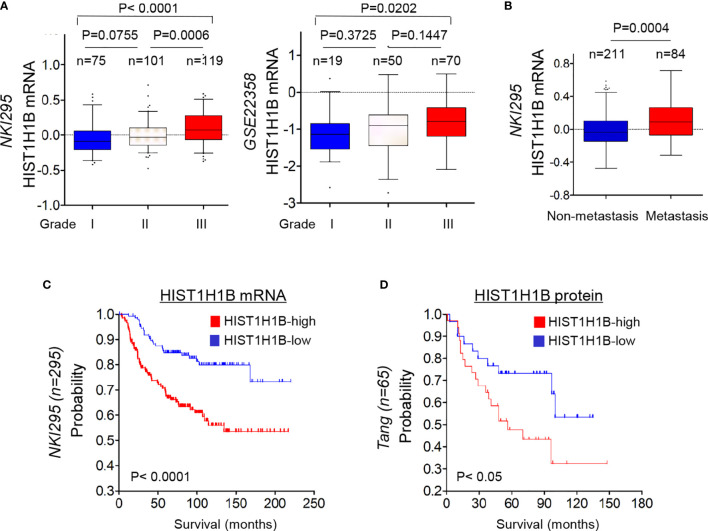
HIST1H1B overexpression associates with higher tumor grade, higher probability of metastasis, and poorer survival of breast cancer patients. **(A)** Box-plots indicated HIST1H1B expression in different histological grades of breast cancer from NKI295 and GSE22358 datasets. Comparisons between two groups are made using the two-tailed Student's t-test. **(B)** Analysis of HIST1H1B expression in breast cancer patients with or without metastasis from the NKI295 dataset. **(C, D)** Kaplan-Meier survival analysis for DMFS of patients in NKI295 and Tang's datasets according to HIST1H1B expression status. The p-value is determined using the log-rank test.

To further explore the clinical implications of HIST1H1B expression for breast cancer progression. We evaluated the relationship between HIST1H1B expression and histological grades of the tumors in NKI295, GSE22358 datasets in which tumors had the grading scores. Patients were segregated into three groups according to tumor grades. Remarkably, HIST1H1B expression was predominantly expressed in high grade, especially in Grade 3 tumors (**Figure 6A**). We also determined the association of HIST1H1B expression with metastasis in the NKI295 dataset, showing a significantly higher probability of metastasis in tumors with high HIST1H1B expression than those with low HIST1H1B expression (**Figure 6B**). We then evaluated the correlation of HIST1H1B expression with patient survival in NKI295 (mRNA level) and Tang’s (protein level) datasets by Kaplan-Meier survival analysis (19). Patients were separated into two groups according to HIST1H1B expression, with high HIST1H1B expression having shorter survival (**Figures 6C, D**). These clinical validations support the critical role of HIST1H1B in breast cancer aggressiveness.

## Discussion

### HIST1H1B Overexpression Is Due to HIST1H1B Copy Number Amplification and Hypomethylation of HIST1H1B Promoter

CNVs in coding gene are critical genetic determinants for some tumor susceptibility (16, 20–24). One possible mechanism that upregulates HIST1H1B expression is an increase in copy number at the genomic locus containing HIST1H1B. In this study, we identified HIST1H1B as a frequently amplified gene by analyzing copy number alteration in the TCGA dataset from breast cancer tissues, and observed that cases with HIST1H1B copy number amplification had much more HIST1H1B expression than ones with no amplification. In addition, in most cases copy number amplification in HIST1H1B and its resultant upregulation of HIST1H1B expression were associated with BLBC. These data strongly suggest that HIST1H1B copy number amplification positively correlates with HIST1H1B expression and BLBC.

Analysis of CNVs reveals that HIST1H1B copy number amplification is only partially responsible for HIST1H1B overexpression in BLBC, suggesting the involvement of other genetic or epigenetic mediators in upregulation of HIST1H1B expression. DNA methylation at CpG dinucleotides has been studied extensively in cancer (25). Because DNA methylation is tightly associated with histone methylation, promoting gene silencing, the DNA hypomethylation may occur due to low enrichment of histone methylation in the promoter of the specific genes (13, 14, 25). The hypomethylation of CpG islands frequently associates with activation of tumor genes, mediating downstream signaling pathways and tumor progression (26). Therefore, high expression of HIST1H1B may be associated with the hypomethylation. Indeed, correlation analysis in gene expression microarray and HIST1H1B methylation datasets demonstrated a negative correlation between HIST1H1B expression and promoter methylation, and the hypomethylation of HIST1H1B specifically occurs in BLBC. Together, the hypomethylation of HIST1H1B promoter is important for the upregulation of HIST1H1B expression.

### HIST1H1B Promotes CSF2 Expression and Tumorigenicity of Breast Cancer

Our study demonstrated a positive correlation between HIST1H1B expression and CSF2 expression. Interestingly, HIST1H1B expression in breast cancer cells significantly promoted CSF2 expressions. We further identified that HIST1H1B was enriched in the ABAT promoter to induce the transcription of CSF2. These data indicate that HIST1H1B is important for upregulation of CSF2 expression. CSF2 upregulation correlates poor prognosis in several tumors by suppressing the immune response and regulating stem cell phenotype (11, 12, 27). It also has been reported that high CSF2 level is a pivotal orchestrator of breast cancer growth (11, 12). Consistent with CSF2 function, knockdown of HIST1H1B expression in BLBC cells remarkably suppressed tumorigenicity *in vitro* and *in vivo*. Together, these data support the critical roles of HIST1H1B -mediated CSF2 expression in the aggressiveness of BLBC.

### HIST1H1B Represents a Potential Prognostic Factor and Therapeutic Target For BLBC

We have identified several factors that might predict patient prognosis, including (1) Breast cancer subtypes: HIST1H1B expression is elevated in BLBC; (2) Tumor size: HIST1H1B overexpression is associated with larger tumor size; (3) Tumor grade: HIST1H1B overexpression is correlated with higher tumor grade; (4) Tumor metastasis: HIST1H1B overexpression has a significantly higher probability of metastasis; (5) Survival rate: HIST1H1B overexpression predicts poor survival in breast cancer patients. These findings strongly support HIST1H1B as a potential prognostic biomarker for breast cancer patients. Treatment of BLBC is a clinical challenge due to the lack of effective targets and drugs. Thus, identifying the new targets in BLBC will be urgently needed. Given the tight association of HIST1H1B with breast cancer aggressiveness, HIST1H1B has the potential to become a therapeutic target of BLBC.

## Conclusions

HIST1H1B expression is upregulated due to HIST1H1B CNV amplification and promoter hypomethylation. HIST1H1B overexpression promotes CSF2 expression and BLBC progression. Our study supports the crucial roles of HIST1H1B in the aggressiveness of BLBC, providing a potential prognostic marker and therapeutic target for BLBC.

## Data Availability Statement

The raw data supporting the conclusions of this article will be made available by the authors, without undue reservation.

## Ethics Statement

The animal study was reviewed and approved by Laboratory Animal Center of Zhejiang University.

## Author Contributions

CD and JL raised conceptions and participated in the design of this research. RL and XC designed and performed most of experiments. QC and YW generated some DNA constructs. ZM and XYL performed some tumorigenesis assays. QJ and JC analyzed gene expressions in breast cancer cells. XW and XLL did the data analysis. CD and JL supervised the work and wrote the manuscript. All authors contributed to the article and approved the submitted version.

## Funding

This work was supported by grants from Natural Science Foundation of China (No. 82173112, 81972456, 81772801 and 81472455 to CD. No. 82103350 to QC; No. 32060163 to XW), and National Key R&D Program of China (No. 2016YFC1303200 to CD).

## Conflict of Interest

Author XL was employed by Abcam Plc.

The remaining authors declare that the research was conducted in the absence of any commercial or financial relationships that could be construed as a potential conflict of interest.

## Publisher’s Note

All claims expressed in this article are solely those of the authors and do not necessarily represent those of their affiliated organizations, or those of the publisher, the editors and the reviewers. Any product that may be evaluated in this article, or claim that may be made by its manufacturer, is not guaranteed or endorsed by the publisher.
